# A Case Series on Beta Thalassemia Major With Secondary Diabetes Mellitus Due to Iron Overload and Other Complications

**DOI:** 10.7759/cureus.93915

**Published:** 2025-10-06

**Authors:** Upendra Prasad Sahu, Neha Rani, Omar Hasan, Naghma Mobin, Praveen Kumar Singh, Shrasta Soumya, Prity K Rajak, Niraj Kumar

**Affiliations:** 1 Department of Pediatrics, Rajendra Institute of Medical Sciences, Ranchi, IND

**Keywords:** beta thalassemia, chelation, diabetes, endocrine dysfunction, hemoglobinopathy, iron overload, rbc transfusion

## Abstract

Beta thalassemia major is an inherited hemoglobinopathy characterized by chronic hemolysis that requires lifelong blood transfusion therapy. With globalization and population migration, it has become a worldwide health concern. Inadequate transfusion therapy can result in complications such as stunted growth, jaundice, hepatosplenomegaly, bone deformities, and extramedullary hematopoiesis. Long-term transfusion treatment frequently leads to iron overload, predisposing patients to diabetes, delayed puberty, and hepatic and cardiac disorders. Additional risk factors, such as family history, lifestyle, obesity, gender, and age, may further increase susceptibility to diabetes in individuals with beta thalassemia.

This case series describes three patients with β-thalassemia major complicated by secondary diabetes mellitus due to iron overload, along with variable multisystem involvement. The patients were managed through an integrated, multidisciplinary approach involving hematology, endocrinology, and cardiology, resulting in clinical stabilization and successful discharge.

This case series highlights the importance of early recognition of iron overload-related complications, including secondary diabetes and associated endocrinopathies, to ensure timely intervention and optimize patient outcomes.

## Introduction

Endocrine dysfunction is very common in beta thalassemia major primarily due to excessive iron deposition in different endocrine glands [1]. There are multiple endocrine functional disorders like growth disruption, delayed pubertal maturation, gonadal dysfunction, endocrine impairments involving the thyroid, parathyroid, and adrenal glands, glucose metabolism disorders such as diabetes mellitus, and skeletal growth abnormalities are also frequently reported [1,2]. Timely diagnosis and the initiation of an appropriate transfusion protocol combined with chelation therapy are critical for effective disease management [1-3].

The standard treatment for thalassemia major involves regular red blood cell transfusions, iron chelation therapy, and management of iron overload-related complications [4,5]. In selected cases, splenectomy may be necessary [5,6]. Currently, hematopoietic stem cell transplantation remains the only definitive curative approach [6]. Significant advancements in transfusion practices, iron chelation regimens, and transplantation techniques have substantially improved the prognosis and quality of life for individuals with β-thalassemia [7]. Despite these improvements, iron overload-induced cardiac disease persists as the primary cause of mortality in affected patients [8]. Thalassemic patients require a multidisciplinary approach to treatment [9].

## Case presentation

Case 1

A 12-year-old boy with a confirmed diagnosis of β-thalassemia major presented with a five-year history of progressive abdominal distension, generalized asthenia for seven days, and a recent onset of exertional dyspnea, non-productive cough, and exercise intolerance over the preceding five days.

On clinical evaluation, the patient exhibited marked pallor, icterus, and phenotypic features consistent with thalassemic facies. Vital signs were within physiological limits. Abdominal examination revealed significant hepatosplenomegaly: the spleen was palpable 10 cm below the left costal margin, and the liver 5 cm below the right costal margin, both firm to palpation, with hepatic tenderness. The cardiac apex beat was displaced inferiorly and laterally to the 6th intercostal space, lateral to the midclavicular line. Auscultation of the precordium revealed a blowing, high-pitched pansystolic murmur at the mitral area with radiation to the axilla, suggestive of mitral regurgitation. Respiratory examination was unremarkable.

Complete blood count demonstrated severe microcytic hypochromic anemia with a hemoglobin concentration of 6.0 g/dL, erythrocyte count of 3.98 × 10^6^/μL, thrombocytopenia with a platelet count of 66 × 10³/μL, and a total leukocyte count of 8.2 × 10³/μL. Red cell indices showed MCV 67.3 fL, MCH 26.5 pg, and MCHC 28.5 g/dL. Peripheral blood smear confirmed a microcytic hypochromic picture. The patient received packed red blood cell transfusions for hematological stabilization.

High-performance liquid chromatography (HPLC) indicated HbF at 88.7%, HbA2 at 3.0%, and HbA at 8.3%, diagnostic of β-thalassemia major.

Liver function tests revealed hyperbilirubinemia with total serum bilirubin of 2.1 mg/dL (direct: 0.8 mg/dL, indirect: 1.3 mg/dL), elevated transaminases (AST: 104 IU/L, ALT: 107 IU/L), and an ALP level of 221 IU/L, consistent with hepatic involvement secondary to chronic hemolysis and iron overload.

Glycemic evaluation demonstrated significant hyperglycemia, with fasting and postprandial blood glucose levels of 357 mg/dL and 510 mg/dL, respectively. In this asymptomatic patient with thalassemia, diabetes mellitus was incidentally diagnosed through elevated fasting and postprandial glucose levels (HbA1c 9.0% being unreliable), and subcutaneous regular insulin was initiated at 1 IU/kg/day in divided doses with dose adjustments guided by capillary glucose monitoring.

Thyroid function assessment revealed overt primary hypothyroidism, with TSH elevated at 12.9 µIU/mL, free T4 decreased at 0.66 ng/dL, and free T3 below the measurable limit (<1.07 pg/mL). Levothyroxine supplementation was commenced.

**Figure 1:**
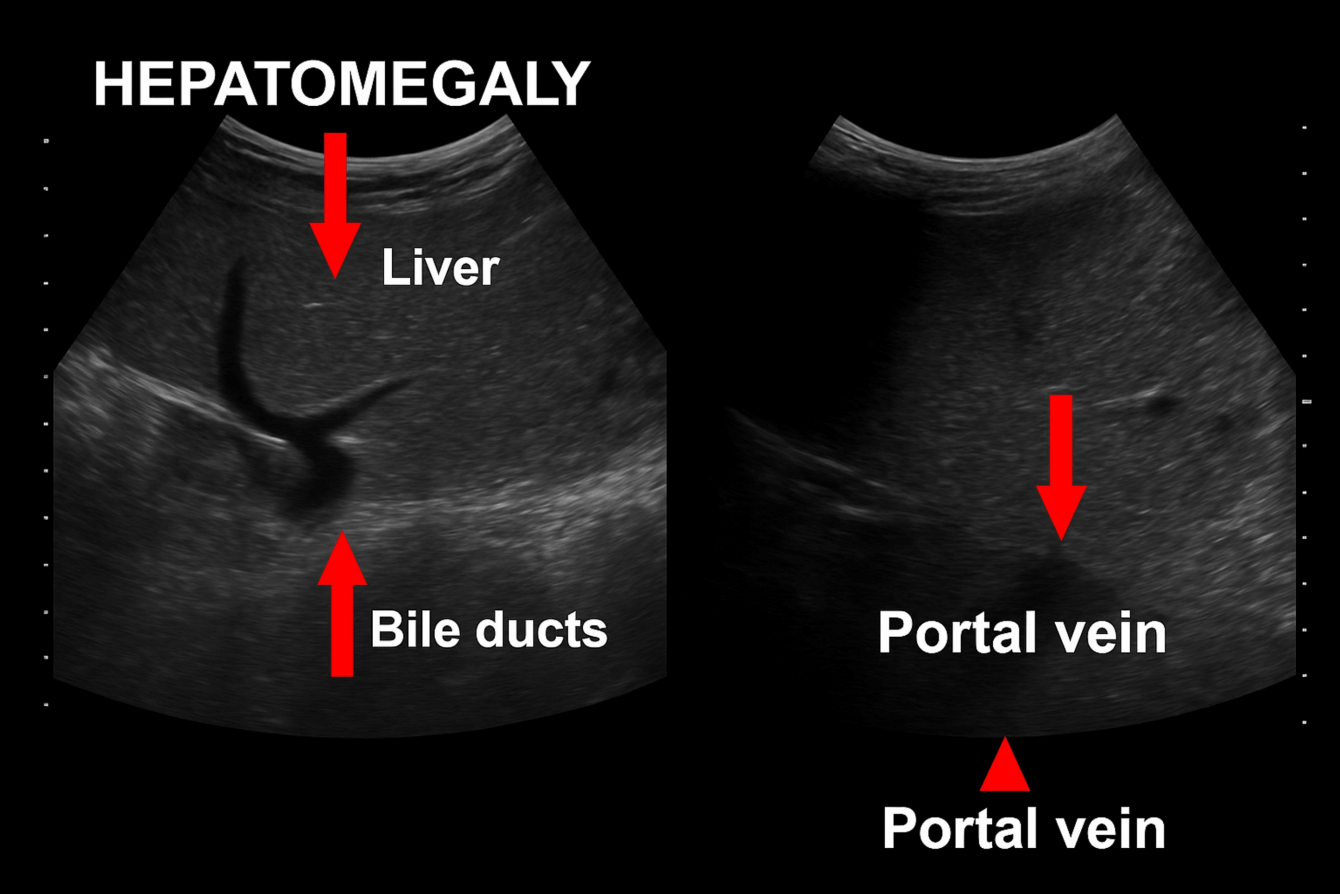
Ultrasonography image of case 1

Serum ferritin was markedly elevated at 6,990 ng/mL, indicative of severe secondary iron overload; chelation therapy was initiated accordingly. Abdominal ultrasonography confirmed hepatosplenomegaly (Figure 1).

**Figure 2:**
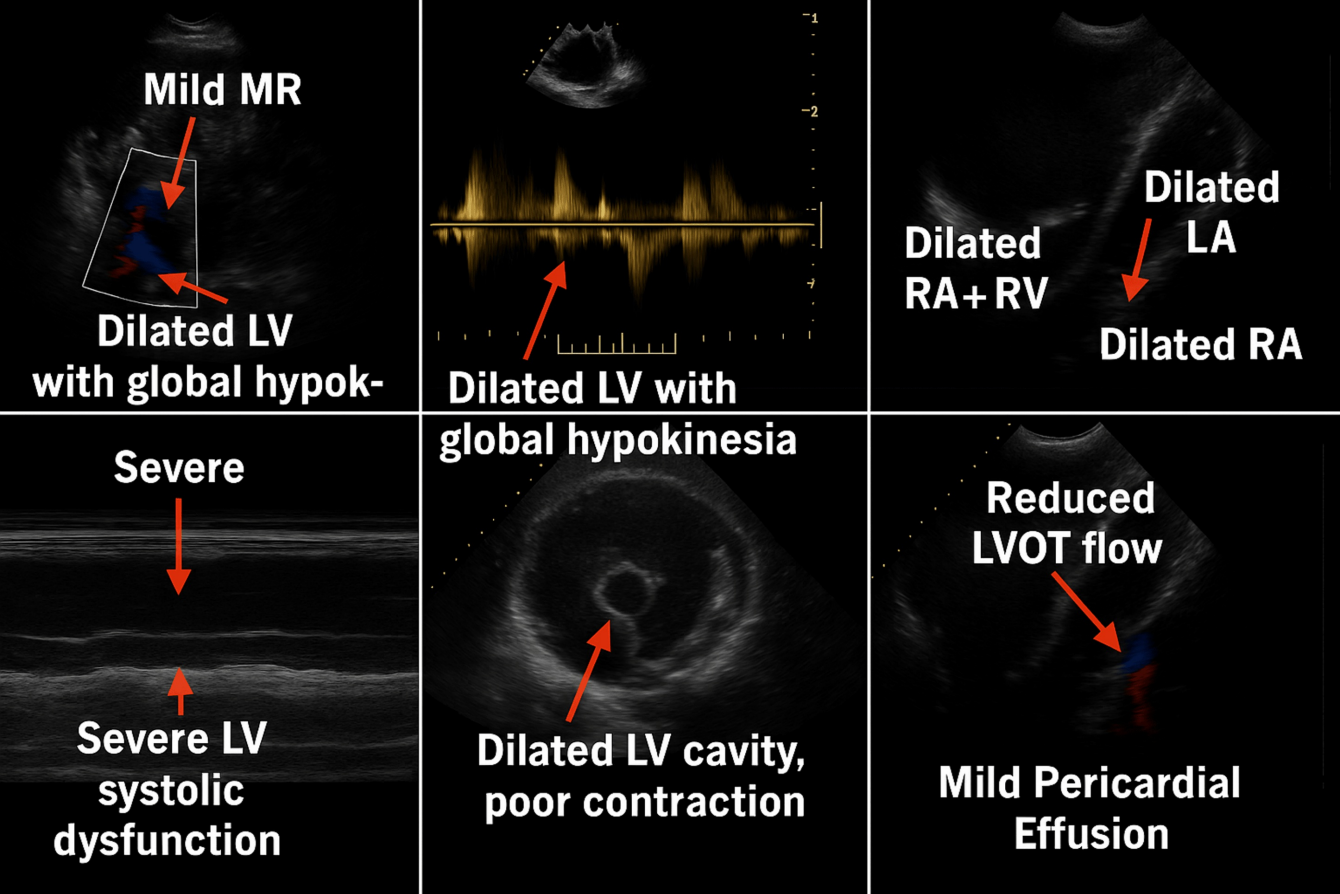
Echocardiography image of case 1 LVOT: Left ventricular outflow tract

Transthoracic two-dimensional echocardiography demonstrated features of advanced cardiomyopathy: global dilation of all cardiac chambers, global left ventricular hypokinesia, a severely depressed left ventricular ejection fraction (LVEF: 20%), grade III left ventricular systolic dysfunction, mild mitral regurgitation, and a mild pericardial effusion (Figure 2). A cardiology consult was obtained, and guideline-directed medical therapy with digoxin, carvedilol, and enalapril was initiated. Table 1 summarizes the laboratory and radiological investigations.

**Table 1:** Lab and radiological investigations of case 1 SC: Subcutaneous

Parameter	Case 1	Reference Range
Hemoglobin (g/dL)	6.0	12.9 – 14.2 g/dl
Platelets (×10³/µL)	66	(155 – 366)×10³/µL
Ferritin (ng/mL)	6990	4.63 – 274.6 ng/ml
HbA1c (%)	9.0	4.0 – 6.0 %
FPG / PPG (mg/dL)	357 / 510	(70 – 100 mg/dl)/(80 – 180 mg/dl)
TSH (µIU/mL)	12.9	0.3500-4.9400Uiu/ml
FT4 (ng/dL)	0.66	0.70-1.48 ng/dl
Echo summary	Dilated cardiomyopathy; global LV hypokinesia; LVEF 20 %; mild MR; mild Pericardial effusion	
Ultrasound abdomen	Hepatosplenomegaly	
Key therapies started	Insulin SC; levothyroxine; iron chelation, digoxin, enalapril, carvedilol, furosemide	

The patient responded favorably to multidisciplinary management involving hematologic, endocrine, and cardiovascular interventions. Following clinical stabilization, he was discharged in an improved condition with recommendations for close, long-term follow-up in a comprehensive care setting.

Case 2

A 14-year-old boy with a confirmed diagnosis of β-thalassemia major presented with a seven-year history of progressive abdominal distension, along with generalized weakness of two weeks’ duration and acute onset of cough, dyspnea, and exercise intolerance for the past seven days.

On physical examination, the patient exhibited severe pallor, icterus, and craniofacial features consistent with thalassemic facies. Additional findings included bilateral pitting pedal edema, tachycardia, and tachypnea. Abdominal examination revealed massive splenomegaly (9 cm below the left costal margin, firm) and hepatomegaly (4 cm below the right costal margin, firm and tender). The apical impulse was displaced inferiorly to the 6th intercostal space, medial to the midclavicular line. Cardiovascular examination identified a high-pitched holosystolic murmur at the lower left sternal border, suggestive of valvular pathology. Respiratory examination was within normal limits.

Laboratory investigations revealed profound microcytic hypochromic anemia with a hemoglobin level of 2.8 g/dL, RBC count of 3.86 × 10^6^/μL, total leukocyte count of 12.8 × 10³/μL, and thrombocytopenia with a platelet count of 91 × 10³/μL. Red cell indices were markedly reduced (MCV: 56.3 fL, MCH: 20.9 pg, MCHC: 28.5 g/dL). Peripheral blood smear confirmed microcytic hypochromic morphology. The patient was transfused with packed red blood cells to achieve hemoglobin optimization.

Hemoglobin analysis by HPLC demonstrated markedly elevated fetal hemoglobin (HbF: 88.3%), reduced HbA (8.9%), and HbA2 (2.8%), consistent with β-thalassemia major. Liver function tests revealed total serum bilirubin of 1.6 mg/dL (direct: 0.4 mg/dL, indirect: 1.2 mg/dL), with serum AST of 60 IU/L, ALT of 49 IU/L, and ALP of 109 IU/L.

Fasting and postprandial blood glucose levels were 187 mg/dL and 208 mg/dL, respectively, with an HbA1c of 7.3%, consistent with newly diagnosed diabetes mellitus; the diagnosis, made as a chance discovery in an asymptomatic patient, led to initiation of subcutaneous regular insulin at 1 IU/kg/day in divided doses, with subsequent titration guided by capillary glucose monitoring. Thyroid function was not reported as abnormal. Serum ferritin was markedly elevated at 7,653 ng/mL, indicative of severe secondary hemosiderosis, and iron chelation therapy was commenced.

**Figure 3:**
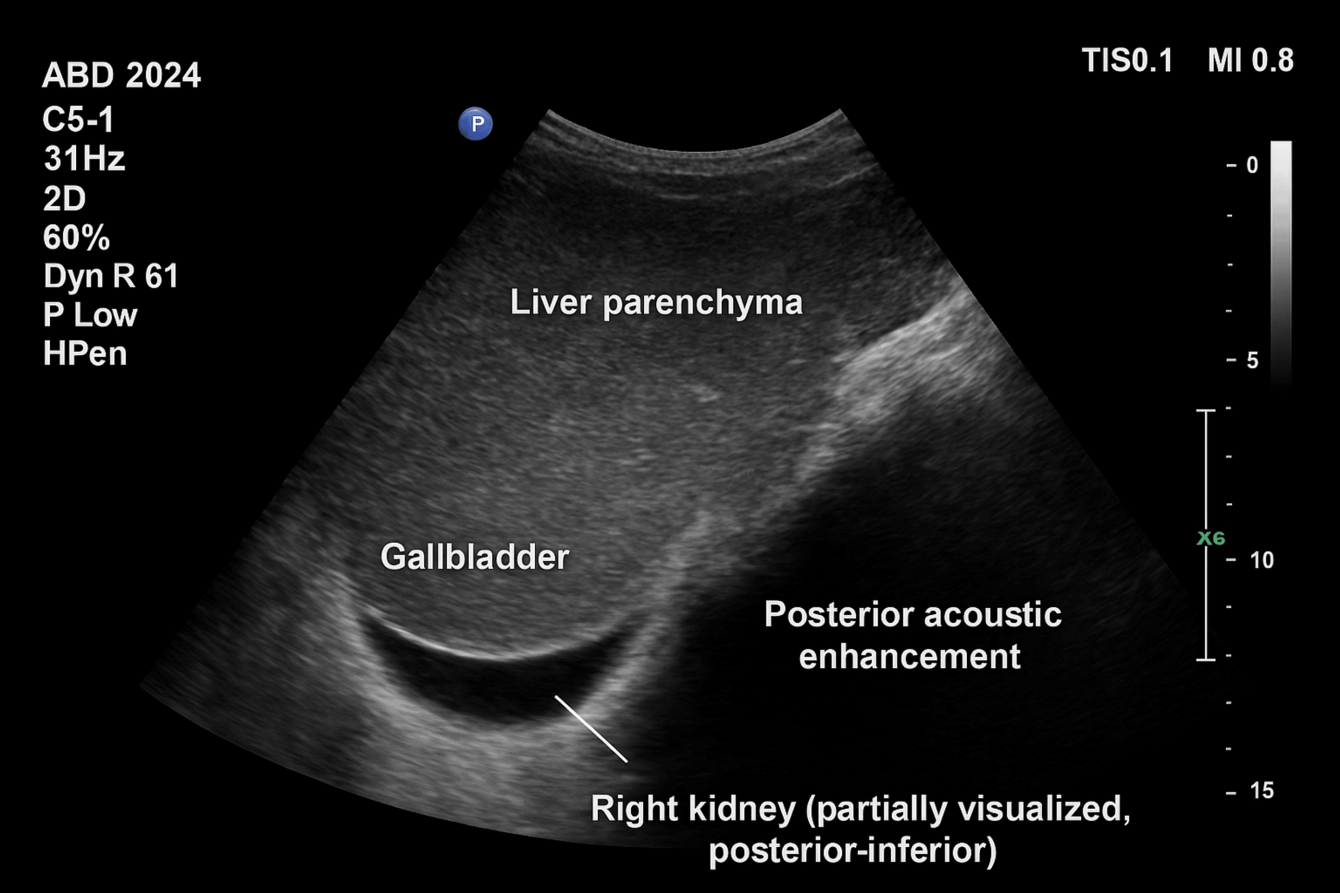
Ultrasonography of case 2

Abdominal ultrasonography confirmed hepatosplenomegaly (Figure 3).

**Figure 4:**
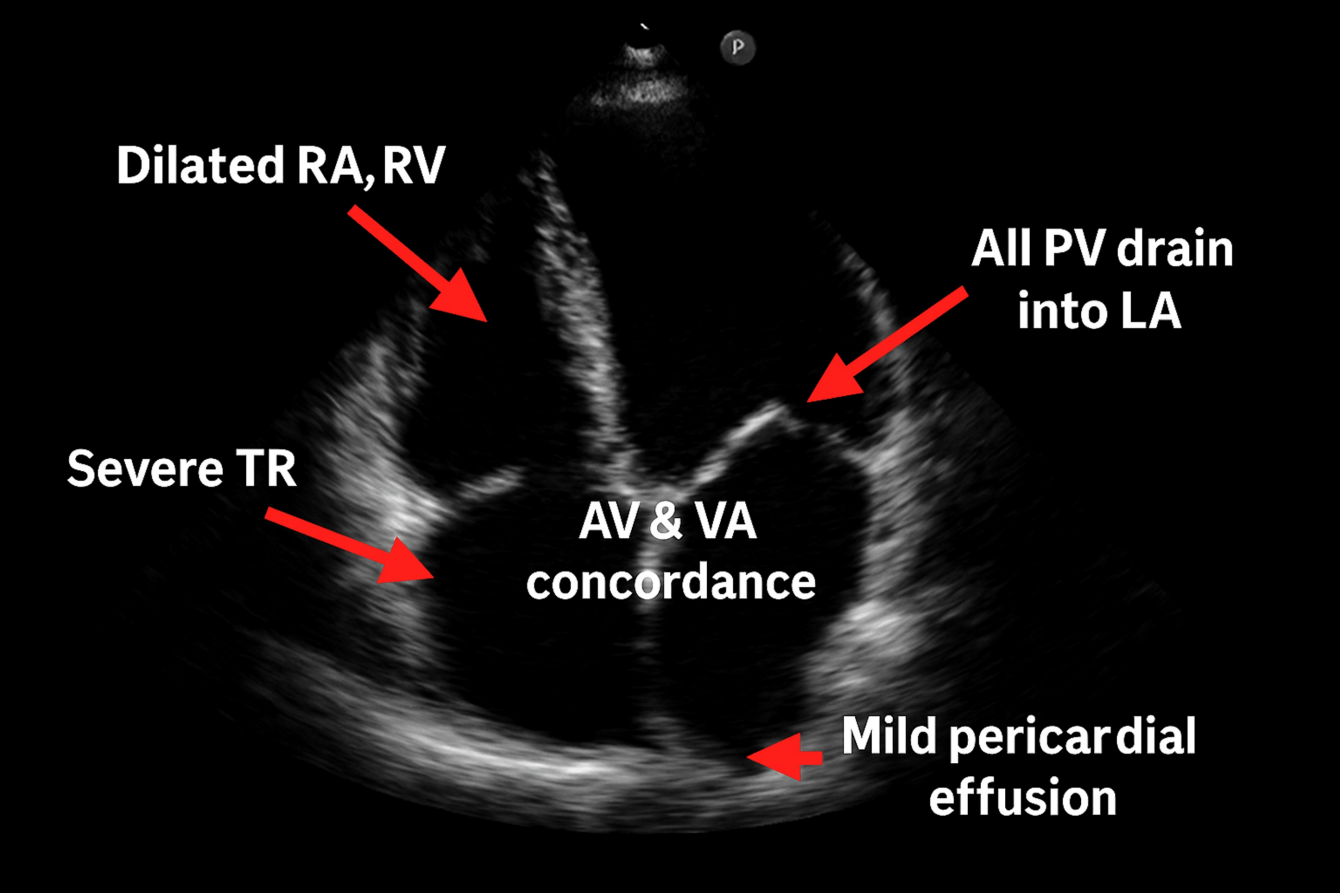
Echocardiography image of case 2 TR: Tricuspid regurgitation

Transthoracic 2D echocardiography revealed significant cardiovascular involvement, with findings of severe tricuspid regurgitation, elevated pulmonary artery pressures consistent with pulmonary hypertension, preserved left ventricular ejection fraction (LVEF: 60%), and right-sided cardiac chamber dilation (right atrium and right ventricle) (Figure 4). Based on cardiology consultation, the patient was initiated on guideline-directed medical therapy including digoxin, carvedilol, and enalapril. Table 2 summarizes the laboratory and radiological investigations.

**Table 2:** Lab and radiological investigations of case 2 SC: Subcutaneous

Parameter	Case 2	Reference Range
Hemoglobin (g/dL)	2.8	12.9 – 14.2 g/dl
Platelets (×10³/µL)	91	(155 – 366)×10³/µL
Ferritin (ng/mL)	7653	4.63 – 274.6 ng/ml
HbA1c (%)	7.3	4.0 – 6.0 %
FPG / PPG (mg/dL)	187/208	(70 – 100 mg/dl)/(80 – 180 mg/dl)
TSH (µIU/mL)	3.8	0.3500-4.9400Uiu/ml
FT4 (ng/dL)	0.9	0.70-1.48 ng/dl
Echo summary	Severe TR; pulmonary HTN; dilated RA/RV; LVEF 60 %	
Ultrasound abdomen	Hepatosplenomegaly	
Key therapies started	Insulin SC, iron chelation, digoxin, enalapril, carvedilol, furosemide	

The patient was managed with supportive care, including transfusion therapy, insulin titration, chelation therapy, and heart failure pharmacotherapy. His clinical condition progressively improved, and he was discharged in stable condition with a multidisciplinary follow-up plan involving hematology, endocrinology, and cardiology services.

Case 3

An 11-year-old boy with a confirmed diagnosis of β-thalassemia major presented with a two-year history of progressive abdominal distension, accompanied by generalized weakness for 10 days and the recent onset of polyuria and polydipsia over the preceding seven days. Clinical evaluation revealed marked pallor, icterus, and characteristic thalassemic facies. Vital signs were within normal limits. Abdominal examination demonstrated hepatosplenomegaly, with the spleen palpable 4 cm below the left costal margin and the liver palpable 3 cm below the right costal margin; both were firm in consistency. Cardiovascular and respiratory examinations were unremarkable.

Hematological analysis showed severe anemia with hemoglobin at 5.8 g/dL, a red blood cell count of 2.98 × 10⁶/μL, platelet count of 106 × 10³/μL, and total leukocyte count of 6.2 × 10³/μL. Red blood cell indices were indicative of microcytic hypochromic anemia (MCV: 71.3 fL, MCH: 26.1 pg, MCHC: 27.5 g/dL). Peripheral blood smear confirmed the microcytic hypochromic morphology. The patient received a transfusion of packed red blood cells to correct the anemia.

Hemoglobin fractionation via HPLC demonstrated elevated HbF (85.7%), low HbA2 (2.3%), and decreased HbA (12%), findings consistent with β-thalassemia major. Liver function tests showed total serum bilirubin of 2.0 mg/dL (direct: 0.8 mg/dL, indirect: 1.2 mg/dL), as well as AST and ALT levels of 49 IU/L and 37 IU/L, respectively, and an alkaline phosphatase level of 121 IU/L.

Fasting and postprandial blood glucose levels were 257 mg/dL and 308 mg/dL, respectively, with an HbA1c of 7.1%, indicative of new-onset diabetes mellitus; led to initiation of subcutaneous regular insulin at 1 IU/kg/day in divided doses, with subsequent titration guided by capillary glucose monitoring. Thyroid function tests were within normal reference ranges. Serum ferritin was markedly elevated at 6,583 ng/mL, suggesting significant iron overload, and iron chelation therapy was commenced.

**Figure 5:**
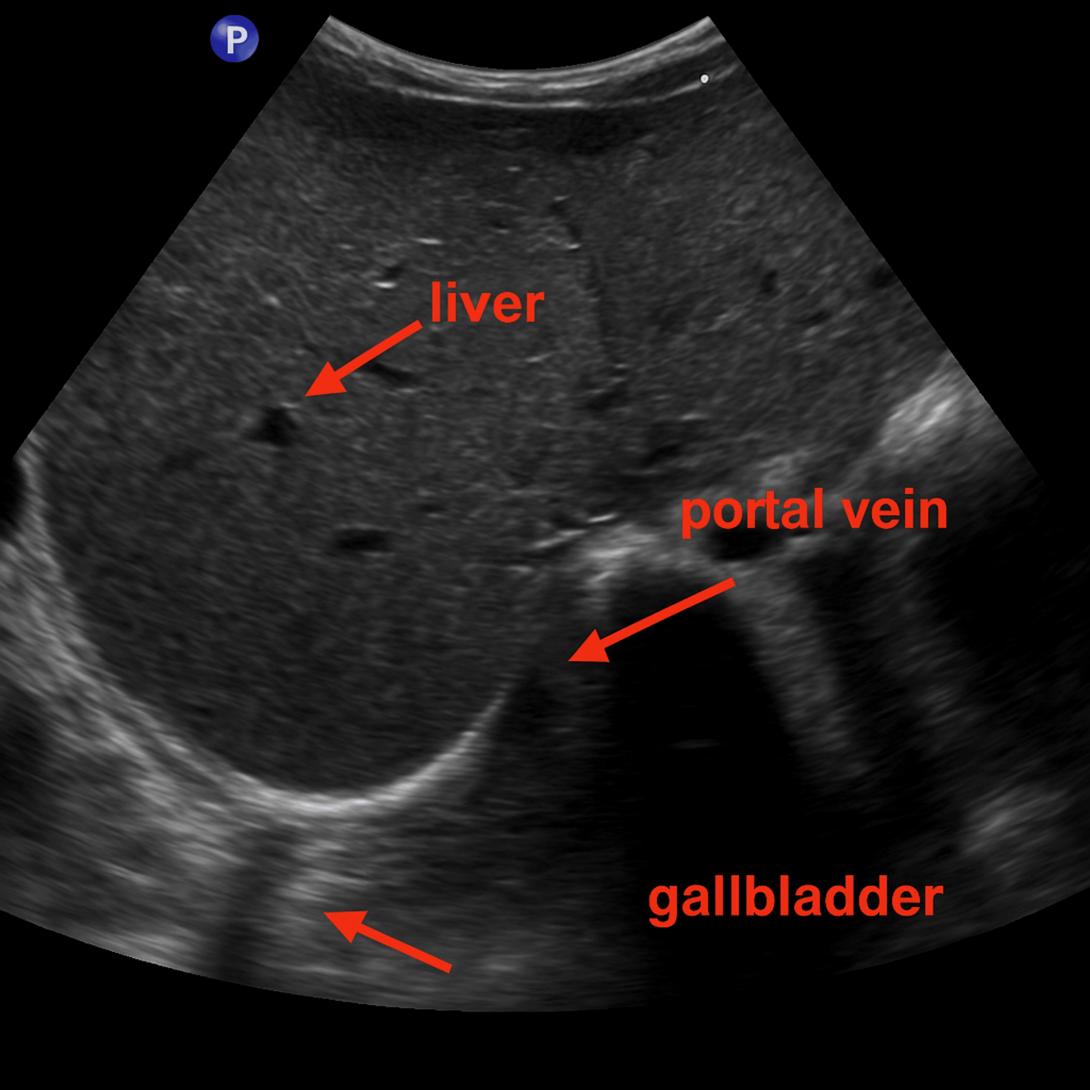
Ultrasonography image of case 3

Abdominal ultrasonography confirmed hepatosplenomegaly without additional abnormalities (Figure 5).

The patient’s blood glucose levels were regularly monitored, and insulin doses were adjusted to maintain euglycemia. With ongoing transfusion support, chelation, and insulin therapy, the patient demonstrated clinical improvement and was discharged in stable condition with multidisciplinary follow-up planned. Table 3 summarizes the laboratory and radiological findings.

**Table 3:** Lab and radiological investigations of case 3 SC: Subcutaneous

Parameter	Case 3	Reference Range
Hemoglobin (g/dL)	5.8	12.9 – 14.2 g/dl
Platelets (×10³/µL)	106	(155 – 366)×10³/µL
Ferritin (ng/mL)	6 583	4.63 – 274.6 ng/ml
HbA1c (%)	7.1	4.0 – 6.0 %
FPG / PPG (mg/dL)	257 / 308	(70 – 100 mg/dl)/(80 – 180 mg/dl)
TSH (µIU/mL)	3.1	0.3500-4.9400Uiu/ml
FT4 (ng/dL)	0.8	0.70-1.48 ng/dl
Ultrasound abdomen	Hepatosplenomegaly	
Key therapies started	Insulin SC; iron chelation	

## Discussion

This case series demonstrates transfusion-related complications of beta thalassemia major, most notably secondary diabetes mellitus due to iron overload (present in all three cases), along with hypothyroidism and cardiomyopathy in one case. These findings emphasize the need for early detection and intervention.

Although type 1 and type 2 diabetes have also been reported in β-thalassemia, our cases highlight the more common secondary form caused by transfusional iron overload. In this report, hyperglycemia was incidentally detected in cases 1 and 2 without symptoms, whereas case 3 presented with symptomatic hyperglycemia.

The cause of these complications can be multifactorial, but it is mainly attributed to iron overload in different organs in these cases [10].

Management requires a multidisciplinary approach involving hematologists, endocrinologists, and cardiologists [11]. The first priority is optimization of hemoglobin levels through timely red blood cell transfusion, followed by aggressive iron chelation to prevent progressive organ damage. Specific endocrinopathies must be addressed with appropriate hormone replacement, while echocardiography-confirmed cardiomyopathy requires standard guideline-directed therapy.

Diabetes mellitus is a prevalent endocrine complication associated with transfusional hemosiderosis and represents a significant source of morbidity [4,12]. This condition arises from multiple contributing factors, including chronic iron overload leading to pancreatic β-cell dysfunction, insulin resistance due to hepatic and endocrine disturbances, and genetic as well as environmental modifiers, with iron overload playing a central role and thereby complicating clinical management [4,13]. It is important to note that HbA1c alone is not a reliable diagnostic tool in thalassemia due to altered red cell turnover and transfusions. In our cases, the diagnosis was supported by fasting and postprandial glucose values and, in one case, the presence of symptoms. Fructosamine testing was not performed due to resource limitations.

Timely intervention, combined with carefully regulated insulin therapy along with early detection of myocardial involvement and optimized iron chelation, may preserve pancreatic function and help prevent cellular damage [4,14,15]. C-peptide levels and islet autoantibody testing were not performed due to lack of availability in our setting; however, none of the patients were on steroid therapy.

As left ventricular diastolic dysfunction typically precedes the onset of systolic dysfunction in the natural course of thalassemia-related cardiomyopathy, its prompt and accurate identification is of paramount importance for guiding timely and effective therapeutic interventions [9,15].

Advanced imaging techniques, such as Doppler echocardiography, are instrumental in assessing progressive myocardial contractile dysfunction; however, their sensitivity remains insufficient for the detection of subclinical diastolic dysfunction at the individual patient level [9,16-18].

Contextualization with international literature

International studies from Mediterranean and Middle Eastern cohorts have reported diabetes prevalence rates of 10-20% among transfusion-dependent β-thalassemia patients [19-21], largely attributed to chronic iron overload and secondary pancreatic dysfunction [22]. Autoimmune diabetes is uncommon in this group, with islet autoantibody positivity reported only rarely [23,24].

Our findings are consistent with these observations, as the likely mechanism in our cases is secondary diabetes from transfusional iron overload rather than autoimmune diabetes. Furthermore, most published data come from high-resource settings; by contrast, our series highlights the diagnostic and management challenges in a resource-limited context, where advanced metabolic and immunological assays are not readily available [25].

## Conclusions

The concurrent occurrence of secondary diabetes mellitus due to iron overload, alongside endocrinopathies such as hypothyroidism, hepatomegaly, and cardiomyopathy, underscores the critical need for early diagnostic surveillance and a multidisciplinary management approach in transfusion-dependent β-thalassemia. Given the limitations of HbA1c as a diagnostic marker in this population, glycemic assessment should rely on fasting and postprandial glucose levels, oral glucose tolerance testing, and, where available, alternative biomarkers such as fructosamine or glycated albumin. Incorporating these parameters into routine follow-up facilitates timely recognition and management of secondary diabetes, thereby improving overall metabolic outcomes.

Cardiovascular involvement remains the principal prognostic determinant and leading cause of mortality in thalassemia syndromes, with myocardial iron deposition serving as the primary driver of heart failure. The coexistence of iron overload, inflammatory, and genetic factors further exacerbates ventricular dysfunction and progression to cardiac failure. Findings from our series highlight the urgent need for tailored, context-appropriate screening protocols, particularly in resource-limited settings where access to advanced metabolic and immunologic testing is constrained.
